# The Double-Edge Sword of Autophagy in Cancer: From Tumor Suppression to Pro-tumor Activity

**DOI:** 10.3389/fonc.2020.578418

**Published:** 2020-10-07

**Authors:** Rodolfo Chavez-Dominguez, Mario Perez-Medina, Jose S. Lopez-Gonzalez, Miriam Galicia-Velasco, Dolores Aguilar-Cazares

**Affiliations:** ^1^Departamento de Enfermedades Cronico-Degenerativas, Instituto Nacional de Enfermedades Respiratorias “Ismael Cosio Villegas”, Mexico City, Mexico; ^2^Posgrado en Ciencias Biologicas, Universidad Nacional Autonoma de Mexico, Mexico City, Mexico; ^3^Laboratorio de Quimioterapia Experimental, Departamento de Bioquímica, Escuela Nacional de Ciencias Biológicas, Instituto Politécnico Nacional, Mexico City, Mexico

**Keywords:** autophagy, cell death, metabolic reprograming, metastasis, carcinogenesis, tumor microenvironment, immune evasion, chemotherapy and targeted therapy resistance

## Abstract

During tumorigenesis, cancer cells are exposed to a wide variety of intrinsic and extrinsic stresses that challenge homeostasis and growth. Cancer cells display activation of distinct mechanisms for adaptation and growth even in the presence of stress. Autophagy is a catabolic mechanism that aides in the degradation of damaged intracellular material and metabolite recycling. This activity helps meet metabolic needs during nutrient deprivation, genotoxic stress, growth factor withdrawal and hypoxia. However, autophagy plays a paradoxical role in tumorigenesis, depending on the stage of tumor development. Early in tumorigenesis, autophagy is a tumor suppressor via degradation of potentially oncogenic molecules. However, in advanced stages, autophagy promotes the survival of tumor cells by ameliorating stress in the microenvironment. These roles of autophagy are intricate due to their interconnection with other distinct cellular pathways. In this review, we present a broad view of the participation of autophagy in distinct phases of tumor development. Moreover, autophagy participation in important cellular processes such as cell death, metabolic reprogramming, metastasis, immune evasion and treatment resistance that all contribute to tumor development, is reviewed. Finally, the contribution of the hypoxic and nutrient deficient tumor microenvironment in regulation of autophagy and these hallmarks for the development of more aggressive tumors is discussed.

## Introduction

Eukaryotic cells, over their lifespan, are continuously exposed to a variety of physical, chemical, and biological stresses that result in homeostatic imbalance. However, cells are equipped with a set of intracellular defense mechanisms to neutralize and adapt to such stress. Macroautophagy, hereafter referred to as autophagy, is an adaptation mechanism to preserve cellular integrity and viability. Intracellular content, including proteins, organelles and portions of cytoplasm, are sequestered in double-membrane structures, called auto phagosomes, that are delivered to lysosomes for degradation of their content (1). Autophagy is strictly regulated by a variety of genes termed autophagy-related genes (ATG). Autophagy in the absence of stress is active at basal levels to degrade damaged cellular components and recycle nutrients to preserve the energetic state of the cell (2). However, in response to stresses, such as nutrient deprivation, hypoxia, genotoxic stress, accumulation of misfolded proteins, inhibition of protein synthesis or presence of pathogens, autophagy is upregulated to maintain cellular homeostasis (1).

Autophagy is dysregulated in distinct pathological conditions, such as infection, aging, neurological disorders and cancer. Autophagy in cancer cells is considered a double-edged sword since, in initial stages of tumorigenesis, it may act as a tumor suppressor by degrading potentially harmful agents or damaged organelles, thus avoiding the spread of damage including DNA alterations (3). However, in advanced stages of tumor development, autophagy is a tumor-promoting mechanism because of its ability to sustain tumor viability in stressful microenvironments. Besides this tumor-promoting activity, autophagy makes a notable contribution to resistance to distinct types of therapy, representing a serious obstacle for successful treatment (4).

According to Hanahan and Weinberg, tumor cells exhibit eight particular characteristics, called as hallmarks of cancer, that include sustained proliferation, evasion of growth suppressing signals, replicative immortality, angiogenesis, immune escape, evasion of cell death, metabolic reprogramming and activation of invasion and metastasis (5). Recent reports demonstrate that autophagy is associated with some of these hallmarks. For example, autophagy and apoptosis are typically considered as opposite pathways, yet under specific biological circumstances, they act in a cooperative fashion for cell demise.

Little is known concerning crosstalk between these pathways in the early stages of cancer development, but an increasing body of evidence suggests that under stressful conditions associated with cancer, autophagy and apoptosis cooperate to limit the growth of incipient tumor cells. Kitanaka et al. reported that autophagy participates in spontaneous regression of high expressing-RAS neuroblastoma. Dying cells during regression do not exhibit morphological and biochemical signs of apoptosis, suggesting that autophagy may serve as an additional mechanism for cell death (6).

Nutrient demand is increased as tumors develop to sustain cell proliferation. Moreover, the uncontrolled proliferation of cells leads to critical fluctuations in the availability of nutrients. Tumor cells display reprogrammed metabolism adapted to stress induced by decreased supplies of essential nutrients. Additionally, some metabolites derived from metabolic reprogramming, activate autophagy to increase recycling of nutrients and sustain tumor viability. Autophagy thus provides tumor cells with metabolic plasticity to tumor cells due to the diversity of substrates degraded (7). The role of autophagy in epithelial to mesenchymal transition as well as during metastasis will also be discussed. Autophagy participates in promoting cell survival against stressful conditions elicited along with these processes (8).

In this review, we will discuss the role of autophagy during tumor development, from early to late stages of tumor growth. Moreover, crosstalk between autophagy and apoptosis, metabolic reprogramming, and metastasis will be examined. Further, the emerging role of autophagy as an immune evasion mechanism is considered. Finally, the repercussions of autophagy in resistance to distinct cancer treatments are assessed.

## Regulation of Autophagy

The mammalian autophagic process can be divided in three phases: phagophore formation, elongation of isolation membranes, and maturation. Under optimal physiological conditions, the nutrient sensor mammalian target of rapamycin (mTOR) interacts with Unc-51-like kinase 1/2 (ULK 1/2) complex, composed of ULK 1/2 kinases, Atg13, Atg101, and FIP200 proteins. mTOR phosphorylates ULK 1/2, causing inhibition of its kinase activity. However, under stress, such as starvation, ULK 1/2 is activated by the kinase of AMP (AMPK). AMPK functions as a monitor of intracellular energy levels by sensing AMP/ATP ratio. During starvation, intracellular levels of AMP increase leading to AMPK activation (9). AMPK regulates activation of ULK 1/2 by direct and indirect mechanisms. The direct mechanism is due to AMPK-mediated phosphorylation of ULK-1 at serine residues 467, 555, and 638, resulting in ULK activation (10). Mutational-directed loss of these residues in ULK-1 in human osteosarcoma U-2 OS cells and mouse embryonic fibroblast (MEF) inhibits autophagy. This loss leads to accumulation of damaged mitochondria (10). The indirect regulation of ULK 1/2 occurs via suppression of mTOR activity. In this sense, AMPK downregulates mTOR by phosphorylation of the tuberous sclerosis complex 2 (TSC2), which is an mTOR inhibitor, or by phosphorylation of the regulatory associated protein of mTOR (Raptor) (11, 12). These post-translational modifications promote mTOR dissociation from the ULK 1/2 complex leading to activation of ULK 1/2 kinase, which phosphorylates Atg13 and FIP200 (1, 11).

**Figure 1:**
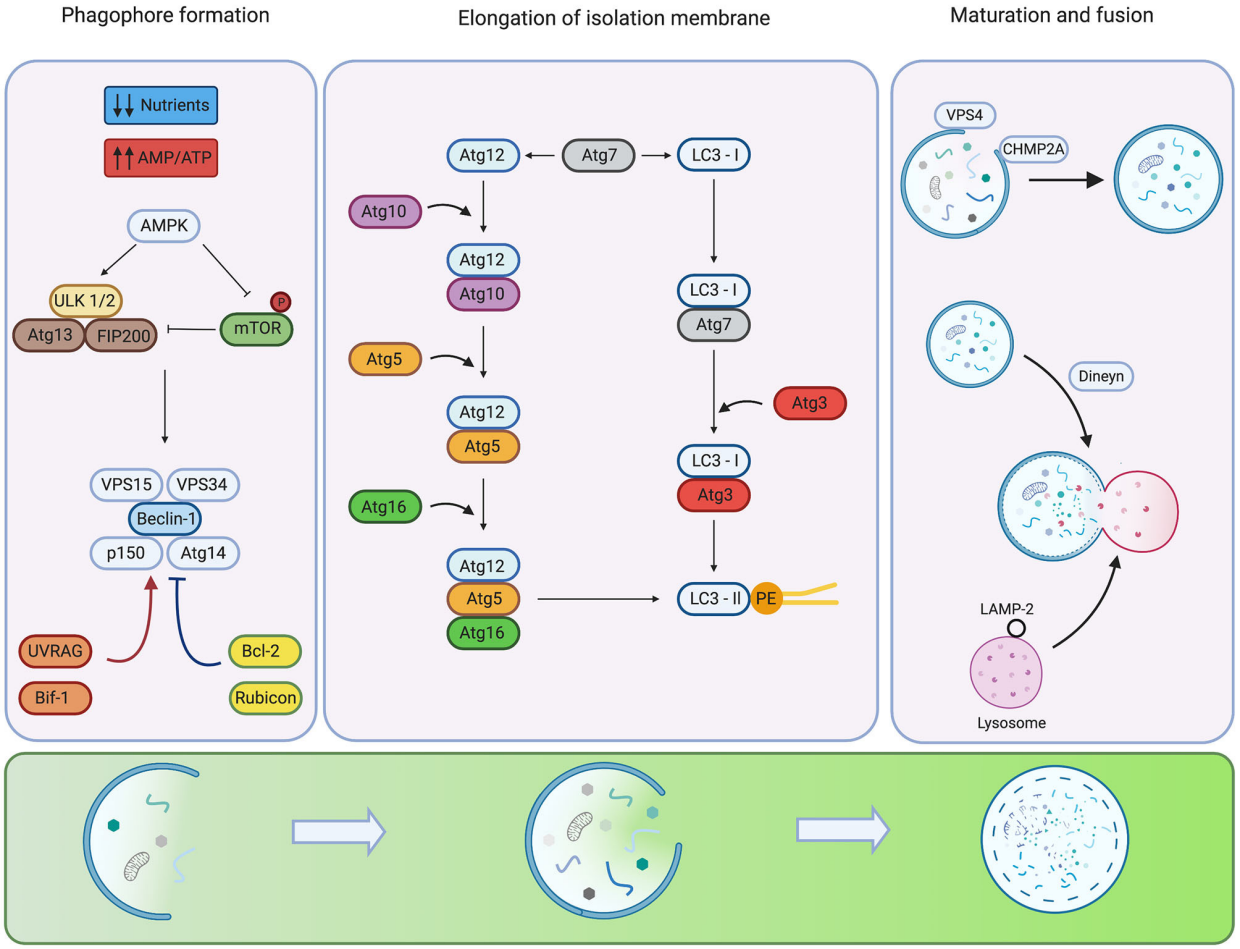
Regulation of the mammalian autophagy. Under low nutrient conditions or starvation, the energy sensor AMPK detects alterations in energy pools (AMP/ATP ratio) inhibiting autophagy repressor mTOR and activating ULK1/2 complex. For phagophore formation, ULK1/2 complex activates the Beclin-1 P13K-class III complex. Additional systems activate (red arrows) or inhibit (blue arrows) the activity and assembly of the complex. The elongation of the isolation membrane requires the participation of two ubiquitin-like conjugation systems. The formation of the Atg12-Atg5-Atg16 complex involves the activity of Atg7 and Atg10. The LC3 requires the participation of Atg4 to hydrolyze LC3 into LC3-I, Atg3 as well as Atg7 for conjugation of LC3-II to pohosphatidyletanolamine (PE). Phagophore closure is regulated by members of ESCRT, CHMP2A VPS4. In the late steps of maturation and fusion, Dynein participates in the mobilization of auto phagosomes. The fusion of auto phagosomes with lysosomes is mediated by members of the SNARE family. Created by BioRender.com.

When ULK-1, located in the nascent phagophore, activates class III phosphatidylinositol 3-kinase (PI3K) VPS34, conversion of phosphatidylinositol to phosphatidylinositol 3-phosphate is promoted by the VPS34, Beclin-1, VPS15, Atg14, and p150 complex (13, 14). The activity of this PI3K complex is modulated in two ways: ultraviolet irradiation resistance-associated gene and BAX-interacting factor 1 (Bif-1) favoring its activity. Conversely, members of the Bcl-2 family, such as Bcl-2 and Bcl-XL, or the run domain Beclin-1 interacting cysteine-rich containing protein (Rubicon), have a negative effect on the activity of the complex (1). In the latter case, Bcl-2 proteins interact with the BH3-binding region of Beclin-1 that prevents their interaction with VPS34, thus inhibiting autophagy. Transgenic mice with Beclin-1 gene mutations in its BH3-binding region show higher levels of basal autophagy in distinct tissues compared to wild type mice (15) (See Figure 1, left panel).

The next step, the elongation of isolation membranes, is regulated by two ubiquitin-like conjugation systems: Atg5-Atg12 and LC3 pathways. The Atg5-Atg12 complex is formed by Atg12 activation by Atg7 and transfer to Atg10 before conjugation with Atg5. Finally, the complex Atg5-Atg12 is non-covalently conjugated to Atg16 to form the complex Atg5-Atg12-Atg16 that displays E3 ligase activity (16). Conversely, the LC3 pathway begins with the C-terminus cleavage of LC3 by the protease, Atg4B, to generate the soluble form, LC3-I. LC3-I is then conjugated to phosphatidylethanolamine (PE) by Atg7, Atg3 and the Atg5-Atg12-Atg16 complex, producing the LC3-II conjugated form (1) (See Figure 1, mid-panel). Some proteins, such as p62 (also known as sequestosome-1), NBR1 and NIX harbor an LC3-interacting region (LIR) which facilitates the recognition of ubiquitylated proteins or specific organelle membranes to selectively deliver cargo to auto phagosomes (17, 18). Although Atg5 and Atg7 are crucial molecules for autophagy, recent studies show that autophagy can be induced by etoposide in Atg5 or Atg7-deficient MEF (19). This Atg5/Atg7 independent form of autophagy is termed “alternative autophagy”. The elongation and closure of the isolation membrane in this alternative pathway are mediated by fusion of endosomal membranes with *trans*-Golgi, and depends on the activity of Rab9 GTPase that replaces Atg5/Atg7 of the canonical pathway (19).

For phagophore closure in the canonical pathway, participation of members of the endosomal sorting complex required for transport (ESCRT), mainly CHMP2A and the vacuolar protein sorting-associated-4 (VPS4), is required (20). CHMP2A is translocated to the edge of phagophore structures in this process to promote closure of the membranes. Also, VPS4 locates on the outer leaf of nascent autophagosomal membranes to promote disassembly of ESCRT molecules in an ATP-dependent manner (20) (See Figure 1, right panel). Experiments carried out in U-2 OS cells demonstrate that genetic inhibition of CHMP2A or VPS4 impairs phagophore closure, preventing the formation of nascent auto phagosomes and causing late fusion with lysosomes (20).

Finally, in the maturation step of auto phagosomes, LC3-II located in the outer autophagosomal membrane is delipidated, and auto phagosomes fuse with lysosomes to form auto phagolysosomes, leading to degradation of auto phagosome content by several hydrolytic enzymes (1). Auto phagosome-lysosome fusion is mainly regulated by soluble NSF attachment protein receptors (SNAREs), specifically Qa-SNARE, syntaxin 17, Qbc-SNARE and lysosomal R-SNARE (21). Also, small GTPase Rab7 and the homotypic fusion and protein sorting participate in auto phagolysosome formation (22) (See Figure 1, right panel).

## Autophagy and Apoptosis Crosstalk in Cancer

Autophagy and apoptosis represent two self-regulatory mechanisms by which cells respond to different types of stresses and death stimuli to maintain homeostasis. Apoptosis is a type of regulated cell death related to the elimination of cells and tissues during embryonic development and also in the removal of damaged cells in adult organisms, thus limiting their proliferation (23). Apoptosis is classified in two mechanisms depending on the type and the source of stress. The intrinsic pathway of apoptosis is activated by intracellular stressors such as DNA damage, endoplasmic reticulum stress, accumulation of reactive oxygen species (ROS), and mitotic defects (23). In contrast, the extrinsic pathway is triggered by extracellular stress and is sensed by distinct death receptors expressed on cell surfaces. Such factors include tumor necrosis factor receptor 1A (TNFR1A) and Fas cell surface receptor (FAS). Activation of extrinsic pathway requires the formation of the death-inducing signaling complex which in turn requires association with TNFRSF1A associated via death domain (TRADD) and Fas-associated via dead domain (FADD) to TNFR or FAS, respectively (23). Both pathways converge in the induction of permeability in the mitochondrial outer membrane, releasing a wide variety of apoptogenic molecules leading to cellular disassembly.

Although autophagy and apoptosis act antagonistically, under specific biological conditions, their crosstalk can lead to cooperation for cellular demise. Currently, accurate molecular interactions of apoptosis-autophagy crosstalk in cancer remain unclear. In the present section, we discuss the participation of key regulatory molecules shared between processes and their impact on cancer, focusing on early stages of tumor development.

**Figure 2:**
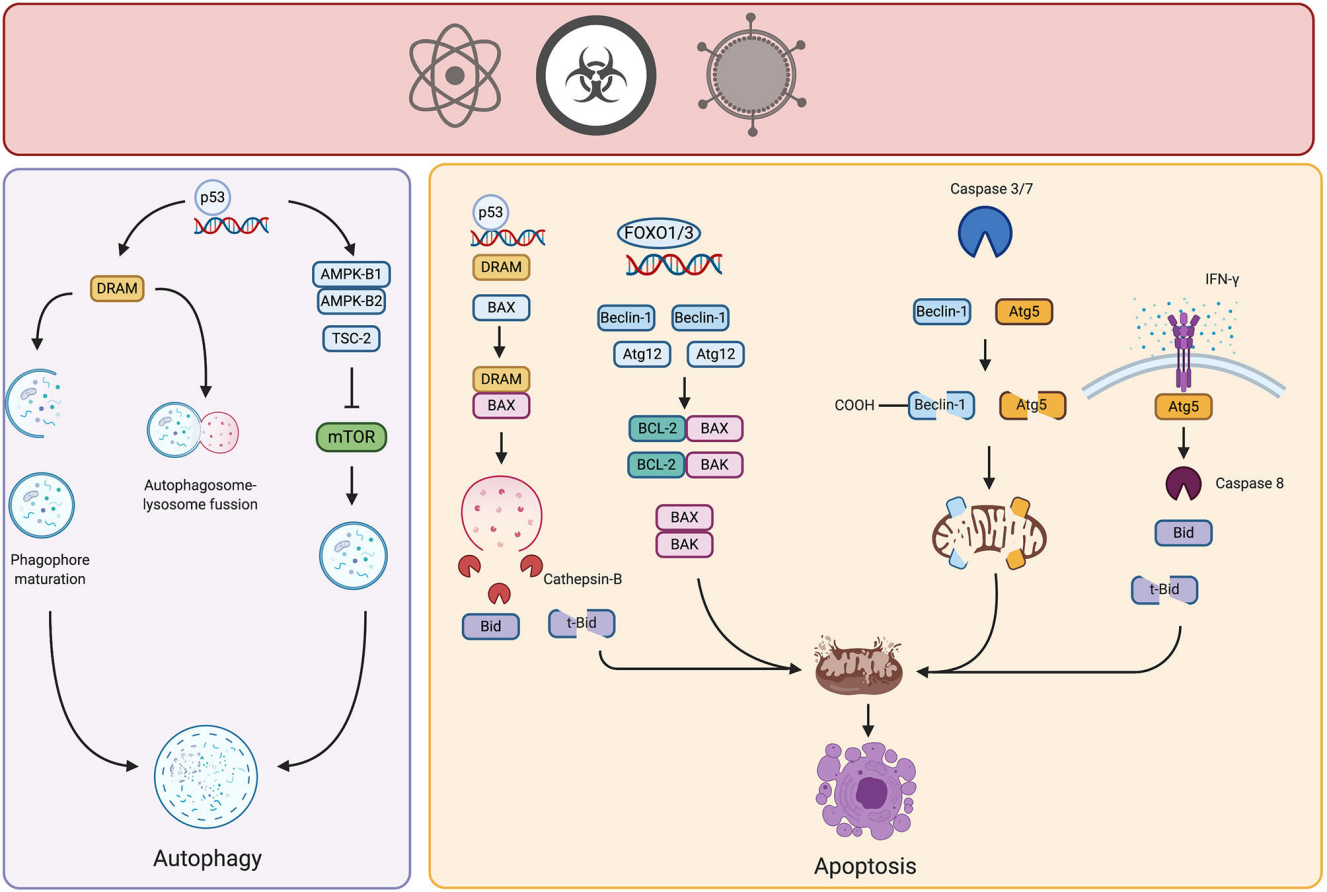
Crosstalk of autophagy and apoptosis in cancer. Potential carcinogenic agents induce distinct types of stress in cell, triggering autophagy or apoptosis. Under certain threshold of damage, stress-responsive transcription factors such as p53 or FOXO promote the upregulation of genes involved in control and activation of autophagy, thereby neutralizing the damage. However, if the carcinogenic stimulus persists and damage is above threshold, autophagic proteins interact with pro- or anti- apoptotic molecules triggering intrinsic or extrinsic apoptosis, therefore limiting the growth of incipient tumor cells. Created by BioRender.com.

As previously mentioned, Beclin-1 is an important protein in the early stages of autophagy. Several studies demonstrate that autophagy may serve as a tumor suppressor. *beclin 1* ^+/−^ mice show a higher incidence of spontaneous lymphomas and carcinomas in lung, liver, and mammary tissue (24). Moreover, Beclin-1 is monoallelically deleted or epigenetically silenced in 50–70% of human breast, prostate and ovarian cancer (4, 25, 26). These findings suggest that Beclin-1 is important for the development of cancer and may serve as a tumor suppressor. Loss of Beclin-1 blocks activation of autophagy, and thus precludes its cytoprotective role. This impairment of degradation of potentially carcinogenic agents or damaged organelles leads to the spreading of damage inside cells and increases the risk of cancer development. In this sense, autophagy is proposed as the “guardian of the genome” since it helps mitigate DNA damage (3). Monoallelic loss of *beclin-1* gene in a mouse model of breast cancer led to increased signs of DNA damage and activity of repair systems, therefore increasing the chance for introduction of mutation and thus the risk of tumorigenesis (27). Besides autophagy, Beclin-1 is implicated in apoptotic cell death, representing a node of crosstalk between these mechanisms (28). *In vitro* experiments show that Beclin-1 overexpression in gastric cancer and glioblastoma cell lines induces apoptosis upon exposure to cytotoxic agents (29, 30). These pro-apoptotic properties of Beclin-1 might be explained by two mechanisms. First, as Beclin-1 interacts through its BH3-only domain with Bcl-2 anti-apoptotic molecules, Beclin-1 overexpression may release pro-apoptotic molecules such as BAX and BAK from Bcl-2 to promote intrinsic apoptosis (Figure 2, right panel). Additionally, caspase-mediated cleavage of Beclin-1 promotes apoptosis. Withdrawal of serum in Ba/F3 murine pro-B cell lines promotes autophagy. However, sustained depletion of growth factors induces apoptosis with activation of caspases which cleave Beclin-1, rendering distinct fragments. The C-terminal fragment moves into mitochondria and introduces and provokes the release of pro-apoptotic molecules, such as cytochrome-c and HtrA2/Omi (31) (Figure 2, right panel). It is possible that in early stages of carcinogenesis, loss of Beclin-1 affects autophagy induction, and also impacts apoptosis regulation, especially in cells with molecular alterations in apoptotic genes.

Members of the Atg5-Atg12-Atg16 complex are also involved in the interplay between autophagy and apoptosis. This complex, as previously mentioned, is part of an ubiquitin-like conjugation system active in the elongation phase of autophagy. Specifically, some findings relate Atg12 protein to apoptotic cell death. Atg12 harbors a BH3-like domain within its structure and physically interacts with anti-apoptotic Bcl-2 molecules such as Mcl-1 and Bcl-2 (32). This interaction may release pro-apoptotic molecules to induce intrinsic apoptosis. For example, Atg12 expression is regulated by distinct transcription factors, such as factors in the forkhead homebox transcription factor family (FOXO) that are induced by different stressors (33). Atg12 is overexpressed after different carcinogenic insults, suggesting that it might participate in autophagy and apoptosis induction in the early stages of carcinogenesis (34). In 2018, Yoo et al. transfected rat intestinal epithelial cells with oncogenic H-RAS and observed that Atg12 was downregulated in these cells due to increased proteasomal degradation, mediated by MAPK activation. In addition, this same group demonstrated that ectopic expression of Atg12 in oncogenic-RAS intestinal epithelial cells resulted in decreased clonogenicity and increased cell death by apoptosis (35). Although increased expression of Atg12 has been found in certain solid tumors, in the early stages of carcinogenesis it might participate in the induction of autophagy also in activation of apoptosis.

*In vitro* studies using HeLa cells indicate that IFN-γ treated cells die by apoptosis preceded by autophagy. Cell death is dependent on expression and interaction of Atg5 and FADD (36) (Figure 2, right panel). Although precise molecular mechanisms remain elusive; the extrinsic pathway of apoptosis is presumably activated. We propose a similar phenomenon in the early stages of carcinogenesis, especially considering the participation of immune response. Immunoediting theory suggests that, during the elimination phase, immune cells remove incipient tumor cells through different mechanisms, involving the release of some cytokines such as IFN-γ (37). Accumulation of this cytokine could lead to the elimination of nascent tumor cells. Moreover, similar to Beclin-1, Atg5 is cleaved by calpain rendering fragments that localize in the mitochondria and promote the release of pro-apoptotic molecules (28).

Another important molecule participating in the crosstalk between autophagy and apoptosis is the BH3-only protein, BIM. BIM interacts with other pro-apoptotic members of the Bcl-2 family during apoptosis to induce the release of apoptogenic molecules from mitochondria, thereby activating the intrinsic pathway (38). BIM is present in cells in three splice variants: BIM-short (BIM_S_), BIM-long (BIM_L_), and BIM-extra-long (BIM_EL_) (39). BIM_S_ and BIM_EL_ participate in apoptosis induction, BIM_L_ displays an important role in autophagy. In IL-7 cultured T-lymphocytes, BIM_L_ localizes in mature lysosomes through interaction with dynein (39). BIM_L_ silencing was not reported, however, lack of BIM_L_ may affect fusion of lysosomes with phagosomes and subsequent degradation of contents. BIM polymorphisms are detected in lung cancer patients (40). We propose that participation of BIM in cancer is crucial since its loss in early stages of carcinogenesis impairs both apoptosis and autophagy, leading to the emergence of tumors.

Another key modulator of autophagy and apoptosis is the tumor suppressor protein TP53, hereafter referred to as p53. p53 is an intracellular sensor of stress caused by genotoxic agents or activation of oncogenes (41). Under non-stressed conditions, p53 is degraded in the cytoplasm by the E3-ubiquitin ligase MDM2. Nonetheless, the cytoplasmic pool of p53 downregulates autophagy by physical interaction with FIP200, thereby inhibiting ULK-1/2 complex activation (42, 43). However, different cellular insults cause stabilization of this protein and localization in the nucleus. In turn, p53 presence in the nucleus leads to upregulation of transcription of distinct genes involved in cell cycle control, repair of damaged DNA, apoptosis and autophagy (41). p53, activated by genotoxic stress, induces autophagy by upregulation of AMPK, thus increasing expression of its β-1 and β-2 subunits and TSC-2, leading to mTOR inhibition, as discussed above (44). In addition, animal models show that the absence of Atg7 induces pancreatic neoplasia without progression to an aggressive phenotype in mice expressing mutated K-RAS. However, the concomitant loss of p53 leads to development of more aggressive pancreatic tumors. Further, p53 activated cell cycle arrest and apoptosis during early stages of tumor development in defective autophagy cells, limits tumor growth (45). These findings suggest that autophagy protects cells from the damage induced by oncogenic signals. Additionally, whether autophagy is defective, p53 limits tumor development by arresting or eliminating incipient tumor cells.

Nuclear p53 also regulates the transcription of the damage-regulated autophagy modulator (DRAM) that represents another point of crosstalk between autophagy and apoptosis. In A549 lung cancer cell lines, soon after exposure to mitochondrial inhibitors or genotoxic agents, DRAM was localized in lysosomes, regulating the process of autophagy in a p53-dependent manner (46). Specifically, DRAM participates in LC3-I to LC3-II conversion, lysosomal acidification, and degradation (46) (Figure 2, right panel). However, sustained stress promotes participation of DRAM in apoptosis, a phenomenon again dependent on p53. Further investigation in lung and cervical cancer cell lines revealed that DRAM regulates apoptosis by disrupting Bcl-2/BAX interaction, interacting with BAX and directing it to lysosomes, where BAX promotes the release of cathepsin-B. Once cathepsin-B is in cytosol, cleaves Bid into t-Bid provoking the release of apoptogenic molecules from mitochondria (47) (Figure 2, right panel). In ovarian cancer, DRAM is downregulated in cell lines and tumor samples of advanced stages, highlighting its participation as a tumor suppressor gene (48). Evidence is poor for participation of DRAM in cancer onset, and we propose that is important in autophagy-dependent clearance of damaged organelles elicited by potentially carcinogenic stimuli sensed by p53, hence, preserving cellular viability. Nonetheless, if carcinogenic stimuli persist or damage is above certain threshold, DRAM might participate in the induction of apoptosis of incipient cancer cells.

Thus, according to the experimental findings and propositions, during early stages of tumor development autophagy and apoptosis cooperate to prevent damage elicited by carcinogenic stimuli or eliminate damaged cells. However, more experimental evidence is required to demonstrate the precise molecular mechanisms governing the crosstalk between these processes during tumor development.

Notably, crosstalk between autophagy and apoptosis in cancer is not steady during tumor progression. Instead, it is modified by intracellular and extracellular perturbations affecting both processes. As tumors evolve, extracellular perturbations caused by a limited influx of nutrients and oxygen modify uptake and metabolism of nutrients and production of intermediary metabolites. Some of these metabolites regulate autophagy activation. Thus, autophagy can be activated via extracellular perturbations, inhibiting cell death, and sustaining cell viability.

## Autophagy in Cancer Metabolic Reprograming

The ability of cells to adapt to stress requires diverse changes in cellular processes, including metabolic pathways. Autophagy is a principal pathway for adaptive metabolic response, an important survival process.

Tumor cells reorganize metabolic pathways to supply ATP, building blocks for macromolecule biosynthesis, and redox molecules required to cell proliferation, invasion, migration, and other processes essential for malignancy, including chemo resistance (49). Consequently, the current research focus on metabolic reprogramming on the development and progression of human cancers reflects these hallmarks of cancer (5, 50).

Otto Heinrich Warburg was the first author to identify changes in the metabolism of tumor cells; he demonstrated that cancer cells avidly consume glucose and excrete high amounts of lactate when oxygen is present. He concluded that tumor cells increase glucose consumption and lactate production because of mitochondrial function (51). This effect was termed the Warburg effect, or aerobic glycolysis (52).

In normal cells, mitochondria oxidize glucose in the presence of oxygen to obtain ATP via the tricarboxylic acid cycle (TCA) and electron transport chain. In the absence of oxygen, the glucose molecule is converted to lactate by lactate dehydrogenase using NADH+, to ensure ATP production and evade glycolysis inhibition. The Warburg effect was initially considered a disadvantage for cancer cells, considering that the amount of ATP produced by the glycolytic pathway much less in comparison to mitochondrial ATP production (53). Nevertheless, glycolysis is the fastest way that cells obtain ATP from the glucose breakdown, and occurs independently of oxygen. Tumor growth is unorganized and the tumor microenvironment is poorly oxygenated; hence, glycolysis allows cancer cells to proliferate even in hypoxic conditions (54). Additionally, this metabolic pathway provides building blocks necessary for other metabolic pathways, such as the synthesis of fatty acids, nucleotides and serine (55, 56).

The Warburg effect is a metabolic adaptation associated with cell transformation that requires oncogene activation, such as RAS, AKT (57), and MYC (58), and the inhibition of tumor suppressors, such as p53 (59, 60). MYC and RAS activation impair decarboxylation of pyruvate, leading to reduce acetyl-CoA production, an essential metabolite in TCA cycle (61). Moreover, in RAS transformed cells, acetyl-CoA production is affected by inhibition of β-oxidation of fatty acids (62). Further, uptake of glucose and glutamine in MYC transformed cells is enhanced along with glycolysis and glutaminolysis (1).

Autophagy supports broad metabolic plasticity to tumor cells, providing biomolecules to almost all carbon metabolic pathways, based on the diversity of substrates degraded (63, 64). For example, the breakdown of several carbohydrates into monosaccharides can fuel glycolysis, and proteins break down into amino acids or degradation of lipids in fatty acids provides substrates necessary for the TCA cycle. This process is essential for metabolic reprogramming (64, 65). Autophagy in tumor cells is closely associated with oncogenic activators and tumor suppressors. RAS activation induces autophagy via PI3K/mTOR, Rac1/JNK, Raf-1/ERK pathways, in addition to the Warburg effect discussed above (63, 66, 67).

Uncontrolled proliferation of malignant cells causes loss of tissue architecture. This structural tissue alteration promotes dysfunctional distribution of nutrients, growth factors, and oxygen within a tumor. Deficient formation of vasculature in the tumor supports the development of heterogeneous tumor microenvironments that differ depending on tumor region (5). The concentration of oxygen is a crucial parameter affected by the heterogeneous nature of tumors. Regions exist where oxygen concentration is <2% within the tumor, therefore, generating a hypoxic zone (68). These hypoxic conditions trigger cellular mechanisms to maintain homeostasis. Hypoxia-inducible factor 1 (HIF-1) is a primary transcriptional regulator during hypoxic conditions. HIF-1 is a complex of two subunits, α and β. The α subunit is degraded under normoxic conditions (oxygen-rich) (69, 70). However, during hypoxia ubiquitylation of the α subunit is decreased, promoting HIF-1 stability. HIF-1 binds to hypoxia-responsive element DNA sequences, facilitating a metabolic shift from oxidative phosphorylation (OXPHOS) to glycolysis (70). In tumor cells, HIF-1 upregulates expression of over 80 genes that are critical in glucose metabolism, cell survival, tumor angiogenesis, invasion, and metastasis, independent of oxygen concentration (71). In hypoxia or starvation, HIF-1 stimulates AMPK and subsequently induces autophagy via BINP3/Beclin-1 or by mTOR inhibition (72). Further, in hypoxia, HIF-1 stimulates transcription of regulated in development and DNA damage response 1 (REDD1) that activates the TSC1/2 complex, thereby inhibiting mTOR activity and promoting autophagy (73). HIF-1 also promotes the transcription of the gene encoding the Bcl-2/adenovirus E1 19 kDa protein-interacting protein 3 (BNIP3) that induces mitochondrial autophagy (mitophagy) by releasing Beclin-1 from Bcl-2 family members, therefore inducing autophagy (69).

However, glycolysis is strictly regulated. The hexokinase (HK) family in mammalian cells catalyzes the conversion of glucose to glucose 6-phosphate (G6P), representing the first rate-limiting step in glycolysis and other metabolic pathways such as pentose phosphate and gluconeogenesis (74). Phosphofructokinase (PFK) is another regulatory enzyme essential in regulating glycolysis. High levels of ATP allosterically inhibit the enzyme, decreasing affinity to fructose 6-phosphate. Thus, ATP/AMP ratio is an essential regulator of PFK. If ATP/AMP ratio is reduced, enzyme activity is increased. In addition, pH also regulates PFK activity. The inhibition of PFK by excessive accumulations of H^+^ prevents the formation and release of lactic acid, which avoids a precipitous drop in blood pH (acidosis) (55).

Nonetheless, overexpression or specific mutations in cancer cells in HK proteins is associated with poor prognosis (75). Specifically, mutations in the catalytic site of PFK enzyme are promoted in the oncogenic process. In glioblastomas, AKT is degraded by polyubiquitylation leading to increased PFK activity, and consequent increase glycolysis, cell proliferation, and tumor growth (76).

Some tumor cells generally express high levels of isoform M2 pyruvate kinase (PKM2) and low levels of isoform M1 of pyruvate kinase (PKM1), a specific regulatory enzyme of glycolysis. Overexpression of PKM1 promotes glycolysis and inhibits mitochondrial oxidative phosphorylation. When PKM2 was knocked out in cancer cells, the PI3K/AKT/mTOR pathway and autophagy were inhibited, thereby leading to a decreased proliferation and inhibition of the invasive phenotype (77). Use of stable isotope tracers (e.g., ^13^C), is currently employed for mapping metabolic pathways. Using this experimental strategy, it is possible to trace the fate of biosynthetic fuels through analysis of downstream isotope enrichment of labeled nutrients. Experiments in cancer patients confirmed that (i) glucose is metabolized through glycolysis and the mitochondrial TCA cycle and (ii) a significant fraction of the acetyl-CoA used in the TCA cycle is not derived from blood-borne glucose (78–80). This information casts doubt on the glycolysis dependency in tumor cells. Besides, accumulating evidence suggests that mitochondrial metabolism is required in tumor cells and is crucial for tumorigenesis, treatment resistance, migration, and metastasis. Some tumors overexpress critical metabolic enzymes and pathways associated with the mitochondrial metabolism. Progression in these tumors is driven by oncogenes and is associated with poor prognosis (52, 74, 81). For example, several cancer mutations in TCA cycle-associated enzymes, such as succinate dehydrogenase, fumarate hydratase, and isocitrate dehydrogenase, contribute to mitochondrial dysfunction during tumorigenesis (82, 83). Autophagy in this case might be essential for providing substrates for anaplerotic reactions, such as amino acids through protein degradation or lipids through turnover, to sustain mitochondrial metabolism (61). Most glucose is consumed by glycolysis, and glutamine becomes the primary substrate for the mitochondrial TCA cycle and generation of fatty acids and NADPH. Autophagy supports necessary metabolic rearrangements which makes cells highly dependent on autophagy for survival.

Metabolites, oxygen concentration, and oncogenes all regulate the initiation of auto phagosome formation, and regulation of autophagy is finely balanced by the integration of these signals. Autophagy is strongly induced in response to nutrient starvation, primarily controlled by mTOR (65).

Glutamine is the most abundant free amino acid and becomes physiologically essential in conditions of high proliferation. Glutaminolysis is the pathway that cells employ to transform glutamine to α-ketoglutarate, an irreversible reaction catalyzed by glutaminase (GLS) and glutamate dehydrogenase. In cancer cells, increased consumption of glutamine has been linked to regulation of oncogenes like MYC. Overexpression of MYC correlates with expression of cellular transporter of glutamine, SLC1A5, and enhances glutamine consumption in cancer cells (84, 85).

Glutaminolysis is proposed as an essential metabolic pathway in tumor cells that supplies carbon for anaplerotic pathways, such as TCA (86, 87). Proliferating cancer cells require high quantities of fatty acids and lipids to generate new membranes. Citrate is diverted from the TCA cycle to sustain fatty acid synthesis, causing TCA cycle disruption, and compelling cancer cells to consume alternative nutrients to reestablish the TCA cycle (87, 88). Hence, glutamine stimulates the production of α-ketoglutarate, reconstituting the TCA cycle. In addition, glutamate produced by GLS is necessary for the synthesis of glutathione (GSH), an intracellular antioxidant that contributes to mitigation of oxidative stress in proliferating cells (88, 89).

α-ketoglutarate, induces translocation of mTORC1 in the lysosome, increasing phosphorylation of ribosomal protein S6 kinase (S6K) and inhibiting the formation of the ULK-1/ATG13/FIP200 complex resulting in inhibition of autophagy (86). However, in cancer cells this link between mTORC1 and glutaminolysis acts in both directions. Starvation leads to the activation of forkhead box O3 (FOXO3), which in turn, increases the expression of glutamate-ammonia ligase, the enzyme that resynthesizes glutamine from glutamate. The increase in glutamine synthesis abolishes the production of α-ketoglutarate from glutaminolysis, and thus inhibits mTORC1 and enhances autophagy (86, 90).

However, the interaction between glutaminolysis and mTORC1/autophagy seems to be more complex. α-ketoglutarate might activate mTORC1 and inhibit autophagy through an alternative mechanism involving acetyl-CoA synthesis and protein acetylation (91). Further, despite the inhibitory effect of glutaminolysis on autophagy, a by-product of glutaminolysis, ammonium, has a dual role in autophagy, activating this process at low concentrations and inhibiting it at higher concentrations (92).

**Figure 3:**
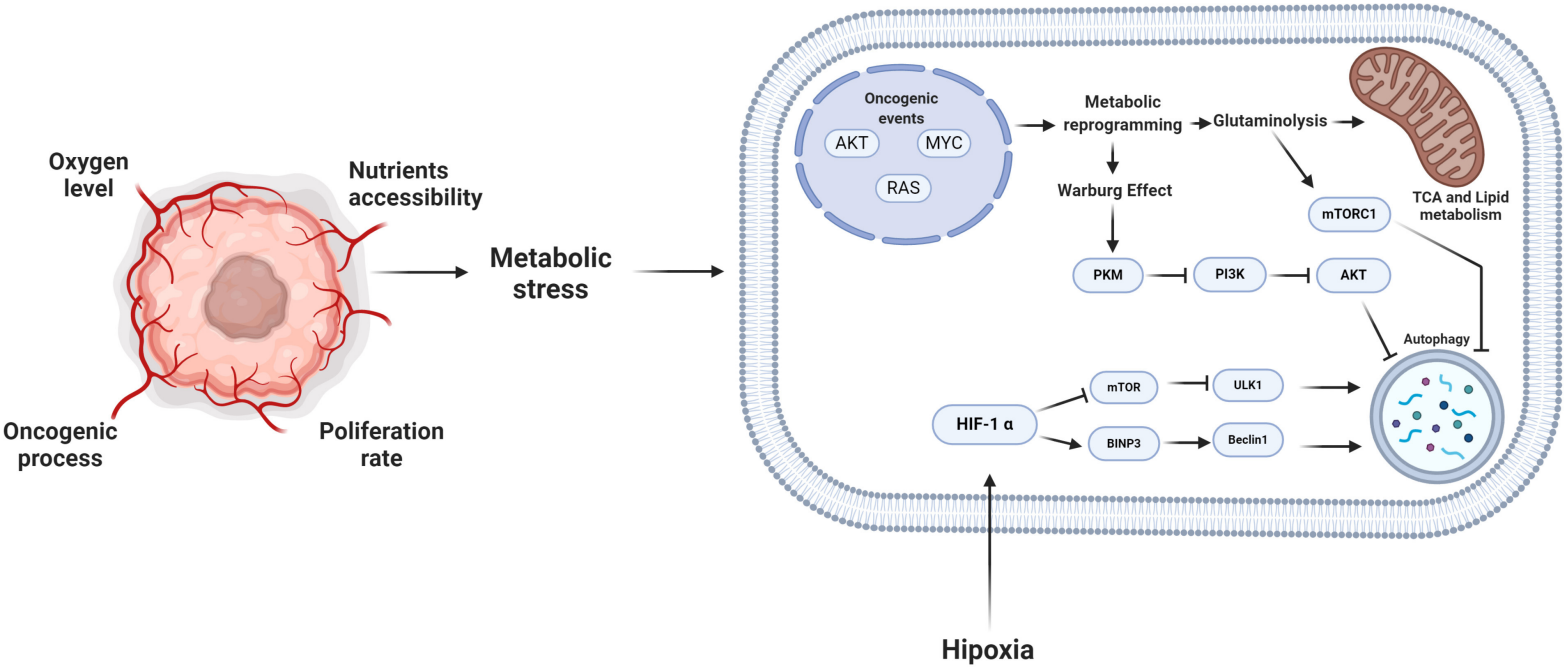
Metabolic stress and autophagy. During the oncogenic process, the proliferation rate and the microenvironmental conditions promote that the tumor cells reprogram their metabolism. Consequently, autophagy plays an essential role in this reprogramming, providing different substrates to feed the pathways of tumor cells. However, the induction of autophagy depends on the stimuli to which the cell is subjected, the alteration of oncogenes such as MYC or RAS, the autophagy process is inhibited and during some microenvironmental tumor conditions such as hypoxia, autophagy is promoted. Created by BioRender.com.

Reprogramming of glucose and amino acid metabolism is accompanied by alterations in lipid metabolism in tumor cells to meet energy demands for sustaining viability and proliferation (Figure 3).

Lipids represent a wide variety of molecules, including sterols, triacylglycerols, and phospholipids. When energy supplies are plentiful, lipids are stored in cells as lipid droplets (LD) to avoid the accumulation of fatty acids in the cytosol (93). However, starvation promotes degradation of lipids stored in LD into fatty acids that are then metabolized by β-oxidation to obtain large amounts of ATP. Two primary metabolic pathways for lipid degradation within LD: neutral lipolysis and autophagic degradation. Neutral lipolysis involves the breakdown of lipids into fatty acids by cytosolic lipases which function under neutral pH environments (94). In contrast, autophagic degradation of LD (termed lipophagy) involves sequestration of portions or entire LD into auto phagosomes with subsequent degradation in lysosomes by acidic lipases (95). Lipophagy was firstly detected and studied in hepatocytes of starved mice (96). More recently, the process was shown in starved adipocytes, neurons and immune cells (96, 97).

Lipophagy is also strictly regulated by a variety of transcription factors that respond to nutrient status, such as the nuclear receptors of farnesoid X receptors, master regulator of lysosomal biogenesis transcription factor EB (TFEB), TFE3, members of the FOXO family and CCAAT enhancer binding protein α (C/EBP-α) (94, 98). The precise molecular mechanism of lipophagy is not clear. It is initiated by recognition of LD mediated by p62, NBR1, and NDP52, which display LIR domains and interact with LC3-II present in phagophores (94).

The role of lipophagy in cancer is still unknown, since some studies report a positive effect in tumor progression and others a negative impact. In 2015, Lu et al. reported that increased expression of C/EBP-α correlated with poor prognosis in patients with hepatocellular carcinoma (98). Hepatocarcinoma cell lines deprived of glucose and glutamine overexpress C/EBP-α and avoid cell death owing to increased lipid catabolism. Further, fatty acid β-oxidation or autophagy inhibition, induced cell death after nutrient deprivation, suggesting that lipophagy protects tumor cells from starvation (98). However, contrasting results were obtained in lung and hepatic tissue of knockout lysosomal acid lipase (LAL) mice that develop more tumors than wild type counterparts and display major susceptibility to metastasis. Further, the absence of LAL was associated with increased release of tumor-promoting cytokines (99, 100). In this case, it seems that lipophagy could act as a tumor suppressor in the early stages of tumor development, and in advanced stages, in which environmental and metabolic alterations are present, lipophagy may promote tumor progression. More studies are required to test this hypothesis.

The metabolic implications of this process are profound and multifaceted. First, autophagy-mediated degradation and recycling of cell substrates supports metabolism and promotes survival and tumor growth. Second, activation of autophagy in response to cancer therapy potentially leads to tumors resistance to conventional chemotherapy.

## The Interplay of Autophagy and Metastasis

Metastasis is a specific process of tumor aggressiveness, and most cancer patients die as a result of metastasis. Metastasis is a response to the challenge of metabolic alterations and tumor microenvironment (101). The unfavorable conditions in this microenvironment, such as hypoxia and lack of nutrients that occur during uncontrolled cell proliferation contribute to the development of metastasis (102). Clear evidence exists of migration of tumor cells at early stages of tumor development, but the metastatic process is associated with advanced stages of tumors. Autophagy plays an essential role in the metastasis cascade (8).

The steps of this cascade are the invasion of tumor cells into the primary site, the intravasation, and survival of the tumor cells in blood or lymph, and finally, extravasation and colonization by tumor cells at a distant site. Studies on the role of autophagy during the metastatic process are contradictory. Autophagy is reported to stop tumor cell metastasis (103, 104), but other authors suggest that autophagy favors metastasis (105, 106). Molecules involved in autophagic process are upregulated during metastasis. The LC3B protein is increased in lymph nodes of breast cancer patients compared to the primary tumor, and the expression of LC3B increases in advanced stages of disease (107). LC3B also increases in metastases of melanoma and hepatocellular carcinoma compared to primary tumors (108). Expression of autophagic molecules DRAM1 and p62 in glioblastoma correlates with a poor prognosis (109). Other molecules with oncogenic activity, such as long non-coding RNA (lncRNA) MALAT1 in pancreatic cancer, increase autophagy during the metastatic process (110). Blocking the expression of PD-L2 in osteosarcoma inhibits LC3-II and Beclin-1, impeding the ability of tumor cells to invade surrounding tissue (111). Annexin-A1 protein inhibits autophagy by activating the AKT pathway, which inhibits ERK-1/2 in nasopharyngeal carcinoma (112).

As previously mentioned, hypoxia is an autophagic-inducing factor, but may also promote autophagy and cell migration. IncRNA CPS1-IT1 in colorectal carcinoma suppresses expression of HIF-1α and decreases epithelium-mesenchymal transition (EMT). Autophagy was observed in this study (113). Levels of BNIP3, PI3KC3, and LC3-II were increased in a model of CoCl_2_-induced hypoxia in cholangiocarcinoma. CoCl_2_ at 100 μM, accelerated cell migration due to upregulation of the metastasis marker, phosphorylated focal adhesion kinase (pFAK) (114).

Soluble factors in the tumor microenvironment, secreted in an autocrine or paracrine manner by the tumor cells, trigger metastasis, and autophagy (115). One such factor is transforming growth factor (TGF)-β. Exposure to TGF-β in non-small cell lung carcinoma cell lines, induced autophagy and EMT (116). Autophagy and EMT are initiated in a TGF-β dependent manner in starved hepatocellular carcinoma cells (117).

The metastasis process begins with tumor cell invasion at the primary site and is coupled with EMT. Neoplastic cells lose adhesion and contact with other cells because of the EMT program (118). Loss of adhesion and activation of EMT trigger cell death stimuli that are avoided by activation of autophagy (119). Autophagy is reported to be mainly involved in promoting cancer cell motility. Tumor cells must evade anoikis, a type of programmed cell death that occurs when a cell detaches from the extracellular matrix. This process of cell death is mediated by apoptosis. Tumor cells can evade anoikis by activating autophagy (120). Another mechanism involving autophagy during cell motility is the degradation of adhesion molecules, such as paxillin in auto phagosomes (8).

### Autophagy and Anoikis

Interaction between cells and extracellular matrices (ECM) requires complex bonds called focal adhesions (FA) (121). These junctions connect the cytoskeleton of epithelial cells with components of the ECM through integrins. On the extracellular side, integrins bind to ECM components, such as collagen, fibronectin, vitronectin, and laminin (122). While in the interior of the cell, the integrins bind to the cytoskeleton by means of a protein complex formed by talin, vinculin, paxillin, zyxin, and α-actin (121, 123). FA is regulated by the focal adhesion kinase (FAK)-Src, which is part of this complex. FA bond composition varies among tissues and recognizes components of ECM, changes in the cell surface, and physiological and mechanical stress. Dissociation of FA from ECM leads to cell death by apoptosis in a process called anoikis (124). The disruption of integrins interactions with ECM activates FAK-Src, which suppresses survival signals such as ERK, PTEN, and NF-kB (125). Lack of cell adhesion activates Bid and Bim, pro-apoptotic molecules that promote the assembly of BAX-BAK oligomers on the outer mitochondrial membrane, activating the intrinsic apoptosis pathway (125). Death by anoikis might also occur via the extrinsic pathway since the loss of adhesion leads to downregulation of FLIP and increased expression of Fas and FasL (125).

The multi-functionality of FA allows detection of reduced integrin signaling that occurs after tumor cell detachment to the ECM. The signal of cell detachment is translated as a signal of metabolic stress, activating pathways such as PI3K-AKT, which has a fundamental role in the regulation of integrins by growth factors such as epidermal growth factor and TGF-β. These signals mediate a cellular survival response and inhibit pro-apoptotic proteins such as Bad, caspase-9, and glycogen synthase kinase 3b, among others (104, 126).

**Figure 4:**
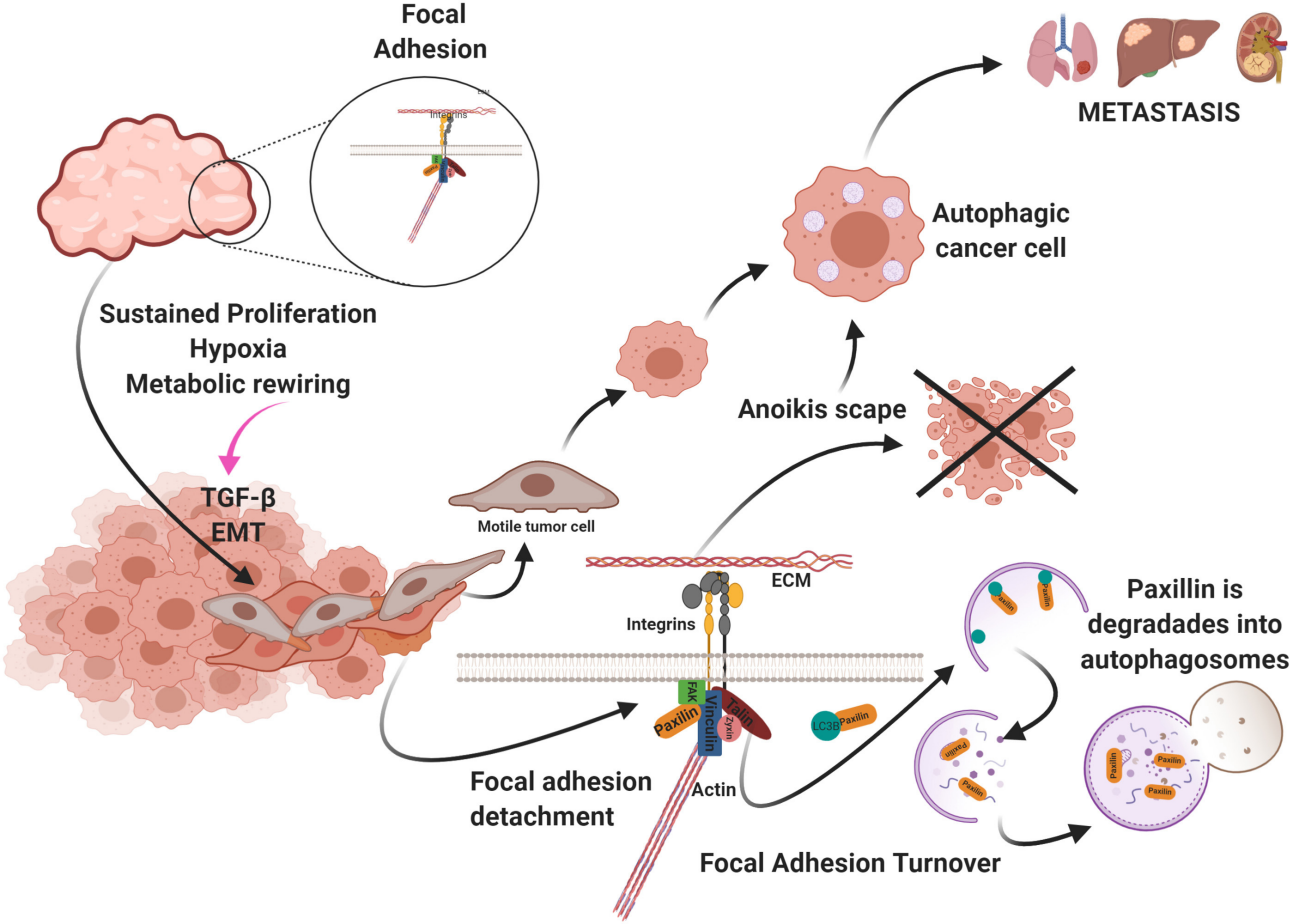
Uncontrolled cell proliferation produces a high demand for oxygen and nutrients. As a result, the tumor becomes hypoxic and starved. These metabolic changes generate the activation of the epithelium-mesenchymal transition (EMT) program and the presence in the environment of factors that promotes metastasis and autophagy such as TGF-β. Autophagy participates in two ways favoring cellular migration: (a) avoiding anoikis and (b) in the turnover of the focal adhesion. Created by BioRender.com.

Tumor cells are remarkably resistant to anoikis, which favors cell motility and metastasis. Autophagy is the primary mechanism of resistance to anoikis in cancer (125). Fung et al. (127), showed in a 3D oncogenesis model of breast epithelial cultures, that cell shedding from ECM induces autophagy and tumor cell survival. In hepatocellular carcinoma cells, cell detachment from the ECM produced inactivation of the mTORC1 complex and activation of autophagy, evading anoikis. BNIP3 was upregulated by the ERK/HIF-1α pathway in this study, leading to autophagy (128). Also, astrocyte elevated gene 1 (AEG-1) protein has a high correlation with metastasis in hepatocarcinoma. AEG-1 induces resistance to anoikis by activating autophagy (129). Another molecule that induces resistance to anoikis by activating autophagy is miR-30a. By inhibiting this miRNA, a decrease in Beclin-1 and Atg5 was observed, as well as an increase in cell death (130) (Figure 4).

### Autophagy and FA

As previously mentioned, cell-ECM attachments are essential for cell homeostasis. During cell migration, FA is involved in generating tension and traction necessary for cell motility. FA at the front of a cell is employed to anchor the cell to ECM, generating tension required to move the cell. At the rear of the cell, FA must be disassembled to producing advancing movement of the cell. This mechanical movement is termed FA turnover (121, 131, 132).

The metabolic stress produced by the lack of oxygen and nutrients in the tumor and the tumor microenvironment activates cellular motility. Autophagy participates in FA turnover in this context, by degrading paxillin in auto phagosomes and disrupting FA. Sharifi et al. found that inhibiting autophagy suppresses metastasis to the lungs and liver without affecting tumor cell proliferation in a metastatic 4T1 mouse model of breast cancer (8, 133). Paxillin in breast cancer and melanoma metastasis serves as FA scaffolding and contains a LIR. FAK-Src phosphorylates this domain in Y40, and paxillin is activated by LC3B and degraded via autophagy (134). Paxillin is recruited via the c-Cbl cargo receptor and LC3 (135). Finally, endothelial cells around the tumor secrete large amounts of the chemokine CCL5 that induces autophagy in tumor cells that display suppressed androgen receptors in a castration-resistant prostate cancer model. These authors reported co-localization of paxillin in auto phagosomes in metastatic tumor cells, indicating that paxillin is degraded via autophagy, favoring the disassembly of FA and cell motility (136) (Figure 4).

### Autophagy During Colonization

The last step in the metastasis cascade is the colonization of host secondary organs. At this point, metastatic cells show EMT, detachment from ECM, intravasation and extravasation. Metastatic cells must reprogram their metabolism to cope with stress induced by metastasis processes.

Colonization represents a final challenging step for metastatic cells since target organs exhibit distinct environmental conditions from the primary tumor. Moreover, organs display varying environmental and metabolic conditions and exhibit distinct ECM composition, oxygen abundance and nutrient disposition (137).

When reaching host organs, metastatic cells encounter these distinct and hostile microenvironments. Cells do not adapt to these adverse environmental conditions, may enter into a state of dormancy. These dormant cancer cells remain clinically undetectable and progress, causing tumor relapse, and organ failure. Signals responsible for triggering tumor outgrowth and colonization of secondary organs remain unknown, the participation of ECM components and aspects of tumor microenvironments likely play essential roles. Dormant cells are characterized by a reversible growth arrest in G_0_-G_1_ cell cycle phases, reduced metabolism and a stem-cell-like phenotype (138, 139). To survive to this stage, dormant cells activate autophagy. Recent findings of Green et al. showed that autophagy inhibition in dormant breast cancer cells of mice decreased their viability, potential to growth and ability to form lung metastases *in vitro* and *in vivo* (140).

When metastatic cells are able to adapt to distinct environmental conditions, cells display a highly flexible metabolism that allows for colonization and formation of secondary tumor foci.

For example, metastatic cells attempting to invade and colonize lungs must adapt to the acute oxidative environment of these organs. To cope with oxidative toxicity, metastatic cells upregulate the expression of molecules controlling endogenous antioxidant responses, such as glutathione peroxidase 1, superoxide dismutase and peroxiredoxins (141, 142). If these antioxidant defense mechanisms are not sufficient, oxidative damage is generated in organelles. A growing body of evidence shows that accumulation of ROS triggers autophagy through distinct signaling pathways such as inhibition of PI3K-AKT-mTOR, and activation of AMPK and MAPK (143). ROS-activated autophagy promotes degradation of damaged material or organelles (143). In 2013, Peng et al. demonstrated *in vivo* that lung metastases of hepatocellular carcinoma cells exhibit higher levels of autophagy than primary tumors (108). In addition, the same group demonstrated that genetic inhibition of autophagy of highly metastatic hepatocellular carcinoma cells blocked lung colonization potential without changing EMT activation, invasion and migration (144). These findings do not provide information about the redox state of metastatic cells in intact and inhibited autophagy, but autophagy could, in theory, be important for protecting cells against oxidative damage in the lungs.

Another example is the colonization of the liver. The liver is characterized into zones with a varying oxygen gradient and high glucose concentrations, therefore showing hypoxic regions enriched with glucose. In this way, any metastatic cell seeking to establish in the liver must be able to adapt to hypoxic and glucose-rich conditions. Several reports demonstrate that, under hypoxia, HIF-1 upregulates transcription of distinct genes involved in glucose metabolism including, but not limited to, glucose transporters and the enzymes, hexokinase 1/2, lactate dehydrogenase (LDH), enolase 1 and pyruvate dehydrogenase kinase 1 (PDK-1) (145). PDK-1 is a negative regulator of pyruvate dehydrogenase complex, thus reducing the entry of pyruvate to TCA cycle, decreasing mitochondrial activity, and promoting glycolytic metabolism. Kim et al. reported that hypoxia-induced transcriptional upregulation of PDK-1 ensures the glycolytic synthesis of ATP, mitigation of hypoxic ROS production and inhibition of apoptosis (146). Dupuy et al. reported that liver metastases upregulate their glycolytic activity under hypoxia by enhancing the activity of the PDK-1 (147). PDK-1 also regulates autophagy in other cellular settings. Quin et al. reported in acute myeloid leukemia cells that PDK-1 associates with ULK-1 promoting its activation and leading to induction of autophagy (148). Mariño et al. reported, in starved human osteosarcoma cells and in mouse heart tissue, that genetic or pharmacological inhibition of PDK genes resulted in autophagy inhibition (149). Participation of PDK-1 in autophagy induction during liver colonization by metastatic cells has not been studied, and we propose that besides promoting metabolic reprograming, PDK-1 also promotes autophagy as an adaptation mechanism to encourage the survival and colonization of liver by metastatic cells.

## Involvement of Autophagy in Tumor Immune Evasion

In the previous sections, we discussed the evolution of tumor microenvironments and how they sustain most hallmarks of cancer such as tumor growth, metabolic reprogramming, and cell death evasion, invasion and metastasis (5). In this sense, cellular components of the tumor microenvironment like endothelial cells, pericytes, cancer-associated fibroblasts and tumor-infiltrating immune cells play a key role in tumor growth (5).

The immune response is implicated as a key factor during tumor development. According to the cancer immunoediting theory, during early stages of tumor development, the immune system recognizes nascent tumor cells expressing neoantigens on major histocompatibility complex (MHC) molecules, thereby promoting tumor elimination mediated by natural killer (NK) cells or cytotoxic lymphocytes (CTL) (150). However, immune-mediated elimination also represents a selective pressure, and highly immunogenic tumor cells are eliminated while less immunogenic tumor cells survive, avoiding immune recognition and destruction, a feature established as a hallmark of cancer (5, 150). Distinct immune evasion mechanisms have been reported. For instance, decreased expression of death receptors; development of an immunosuppressive microenvironment through release of cytokines, such as TGF-β and IL-10, and recruitment of immunosuppressive cells (150). Emerging evidence also demonstrates that autophagy plays a key role in protecting tumor cells against immune-mediated elimination. In the present section, we discuss the participation of autophagy as an immune evasion strategy, focusing on NK and CTL-mediated elimination.

**Figure 5:**
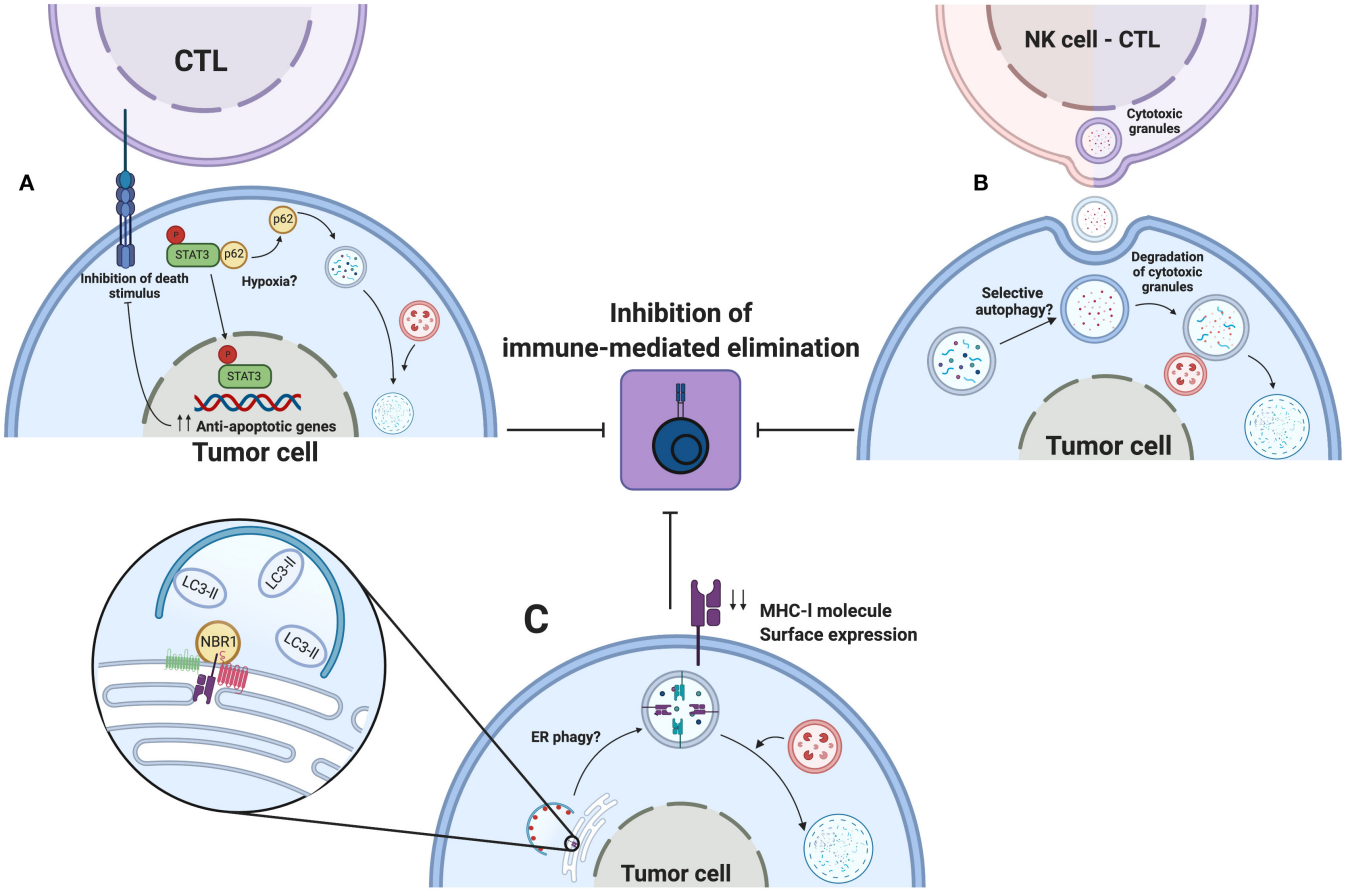
Autophagy as an immune evasion mechanism. Autophagy induced by environmental stress such as hypoxia promotes the escape to CTL or NK mediated elimination of tumors cells. **(A)** Hypoxia, by an undefined mechanism, releases pSTAT3 from p62, thereby degrading p62 by autophagy and favoring pSTAT3 nuclear localization to up-regulate transcription of antiapoptotic genes. **(B)** During hypoxia, tumor cells activate autophagy to sequester and degrade cytotoxic granules released by NK cells or CTLs, thus impeding the elimination of tumor cells. **(C)** Selective ER phagy might participate in degradation of MHC-l molecules during their biogenesis. NBR1 associate to MHC-l molecules or their chaperones in ER. Decreased surface expression of MHC-l molecules leads to impaired recognition by innate or adaptive immune cells, leading to escape for immune-mediated elimination. Created by BioRender.com.

Autophagy is induced in response to adverse conditions elicited by the tumor microenvironment, such as nutrient deprivation and hypoxia. Tumor cells activate autophagy to help meet energy demands and sustain viability and proliferation. Additionally, in 2009 Noman et al. reported that hypoxic conditions impaired elimination of non-small cell lung carcinoma cells by autologous CTL (151). They found that stabilization of HIF-1α and increased phosphorylation of the signal transducer and activator of transcription 3 (pSTAT3), in tumor cells, were associated with evasion of immune surveillance. Further studies performed by this group demonstrate that hypoxia-induced autophagy is responsible for this phenomenon since pharmacologic or genetic inhibition of autophagy in hypoxic conditions restored susceptibility of tumor cells by CTL elimination (152). Further, inhibition of autophagy during hypoxia promoted pSTAT3 degradation in proteasome in a p62-dependent manner. Autophagy degrades p62 and consequently enhances the accumulation of pSTAT3. However, the mechanism by which hypoxia promotes the dissociation of pSTAT3 from p62 remains unclear. Molecular mechanisms are not completely studied, but STAT3 activation by hypoxia-induced autophagy in tumor cells could, in theory, help in escaping CTL-mediated elimination, since this transcription factor controls the expression of anti-apoptotic genes (153) (See Figure 5, upper panel).

Autophagy is also implicated in decreased susceptibility of tumor cells to elimination by NK cells. Baginska et al. reported, in MCF-7 breast cancer cells, that hypoxia-induced autophagy blocked NK cell-mediated lysis of tumor cells (154). In this study, recognition of tumor cells by NK cells and NK cell degranulation were not affected by hypoxia. Instead, tumor cells sequestered granzyme B and perforin granules inside auto phagosomes for subsequent degradation. These findings are supported using *in vivo* models, in which tumor growth of melanoma or breast cancer cells in C57BL/6 or BALB/c mice was reduced in autophagy-deficient tumor cells (154). Results obtained in this work led us to propose that a similar mechanism of cytoprotection elicited by autophagy could be responsible for impaired elimination of tumor cells by CTLs, such as granzyme B and perforin that are also present in CTLs (150) (See Figure 5, upper panel).

Collectively, these findings support the notion that tumor microenvironment has a critical role in tumor development since hypoxic conditions promote the activation of autophagy to protect cells against elimination by innate or adaptive immune cells.

A main aspect during CTL-mediated elimination of tumor cells is the interaction between MHC-I molecules, harboring tumoral neoantigens, and TCR on surface of primed CTL (150). However, tumor cells develop distinct evasion mechanisms to limit this interaction. For example, mutations in the beta-2 microglobulin coding gene or deletions in genes involved in antigen processing are responsible for downregulation of MHC-l molecules (155, 156). Current evidence demonstrates, in pancreatic ductal adenocarcinoma cell lines, that autophagy promotes degradation of MHC-l molecules, therefore reducing their surface expression (157). In this study, MHC-l molecules are targeted for selective autophagic degradation mediated by NBR1. Pharmacologic or genetic inhibition of autophagy increased surface expression of MHC-I molecules and restored susceptibility of pancreatic tumor cells for elimination by CTLs. An increased number of infiltrating CTLs and reduced tumor volume were found using a genetically engineered mouse model (157). Also, concomitant inhibition of autophagy by expression of mutated ATG4B in cancer cells and systemic administration of chloroquine improved efficacy of dual immune checkpoint therapy. This work reveals new insights in the participation of autophagy as an immune evasion strategy, yet some questions remain.

First, MHC-l molecules were degraded by selective autophagy and neither by LC3-associated phagocytosis nor LC3-associated endocytosis, and we speculate that this degradative process occurs during biogenesis in the endoplasmic reticulum. Therefore, NBR1 could interact with MHC-l molecules or their chaperones (calnexin, calreticulin, ERp57), to mediate selective degradation of the endoplasmic reticulum (158) (See Figure 5, lower panel). However, more studies are required to test this possibility.

Second, results were obtained in non-stressful conditions, in which basal levels of autophagy in tumor cells were associated with degradation of MHC-l molecules. However, study during hypoxia, nutrient starvation or other micro environmental stress could determine if these alterations enhance degradation of these and other surface molecules.

Autophagy has a pivotal role in the late stages of tumor development, assisting with immune evasion. This finding points for inhibition of autophagy as a therapeutic alternative to treat tumors.

## Role of Autophagy in Chemotherapy and Target Therapy Resistance

Chemoresistance is the leading challenge anti-tumor therapy, mainly in advanced stages of cancer. Several mechanisms for chemoresistance are recognized, including autophagy. Stress produced by chemotherapy induces autophagy as a cytoprotective mechanism, allowing the tumor cells to resist chemotherapeutic treatment (159, 160).

Cis-diamminedichloroplatinum (II) (cisplatin) is a platinum-based compound approved since the 1970s for the treatment of various neoplasms, such as bladder, ovarian, lung, head and neck, testicular, and others (161). Cisplatin induces autophagy through increased expression of BECN1 in bladder cell lines, which promotes resistance of these cells to the drug (162). Overexpression of thioredoxin-related protein of 14 kDa (TRP14) in ovarian cancer cell lines decreases sensitivity to cisplatin. TRP14 induced autophagy by activating AMPK and inhibiting mTOR and p70S6K. When TRP14 expression was inhibited using shRNA, sensitivity to cisplatin was markedly increased (163). Lung adenocarcinoma cell line A-549/DDP is resistant to cisplatin, and expression of tripartite motif-containing proteins (TRIM)-65 is enhanced along with LC3-II expression. When TRIM65 is inhibited by shRNA in cell lines and in a mouse xenograft model, the cisplatin-induced apoptosis increased, associated with reduction of ATG5, ATG7, and Beclin1 mRNAs levels (164). The LncRNA-small nucleolar RNA host gene 14 (SNHG14) is an antisense sequence of the ubiquitin-protein ligase. In colorectal cancer biopsies, high expression of SNHG14 and ATG14 was observed. In the same work, SNHG14 inhibited miR-186, which blocked ATG14 expression in cisplatin-resistant colorectal cancer cell lines. The authors concluded that SNHG14 induced autophagy and cisplatin resistance by inhibiting miR-186 (165). In another study, cisplatin resistance was related to autophagy by inhibiting the expression of Bcl-2 associated athanogene 3 (BAG3) in cisplatin-resistant ovarian epithelial cancer SKOV3 cells. Autophagy inhibition in SKOV3 cells increased sensitivity to cisplatin (166). In osteosarcoma cell lines, the heat shock chaperone molecule HSP90AA1 is overexpressed when cells are treated with cisplatin, doxorubicin, and methotrexate. Treatments induce autophagy through the PI3K/Akt/mTOR signaling pathway, resulting in resistance to chemotherapy. When HSP90AA1 was inhibited, autophagy was blocked and sensitivity to chemotherapy was enhanced (167). These studies demonstrate that autophagy acts as a cytoprotective mechanism against cytotoxic agents.

Food Drug Administration (FDA)-approved targeted therapy is classified in two groups, monoclonal antibodies, and small inhibitor molecules. These compounds block the growth of tumor cells by interfering with specific and essential molecules required for tumor development (168).

In the breast cancer cell line, MCF-7, which is estrogen receptor-positive and resistant to 4-hydroxytamoxifen (4-OHT), inhibition of autophagy with siRNAs for Atg5 and Beclin-1 increased sensitivity to tamoxifen (169). Further, exposure of MCF-7 cells to 4-OHT induced autophagy in 95% of the cells, yet only 15–20% exhibited markers associated with active cell death II (ACDII). When cells were treated with 4-OHT and 3-methyladenin (3-MA), an inhibitor of auto phagosome formation, or siRNA for Beclin-1, the cells showed sensitivity to 4-OHT (170).

Autophagy is usually a mechanism of resistance for targeted therapy, but contradictory results are reported. Cetuximab is a monoclonal antibody approved by the FDA that inhibits epidermal growth factor receptor (EGFR). Exposure of A431 human vulvar squamous carcinoma, DiFi colorectal carcinoma, HN5, and FaDu head and neck carcinomas cells to cetuximab, elicited diverse responses. In DiFi cells, cetuximab induced cytoprotective autophagy, which was inhibited with chloroquine, thus activating cell death. In A431 cells, cetuximab induced a slight apoptotic response, which was potentiated with an autophagy inhibitor such as chloroquine or activator such as rapamycin. Finally, in HN5 and FaDu cells, cetuximab induced a cytostatic effect. By exposing these cells to a combination of cetuximab and rapamycin, cell death was induced (171).

The antitumoral compounds erlotinib and gefitinib are first-generation tyrosine kinase inhibitors (TKI's) that target cells harboring EGFR-activating mutations, causing growth inhibition and cell death. However, these TKI's trigger cytoprotective autophagy. Cell lines, such as HeLa-R30, are resistant to erlotinib, yet do not display autophagy. When these cells were treated with erlotinib and rapamycin, cell death was increased. The depletion of ATG7 with siRNA restored erlotinib resistance, suggesting that defects in autophagy might be a mechanism of resistance (172). Osimertinib (OSI), is a third-generation EGFR TKI that has been approved for the treatment of NSCLC patients harboring EGFR T790M mutation. Exposure of NSCLC cell lines H-1975, HCC827, and A-549 to OSI induced ROS, which in turn activates autophagy leading to decreased cell viability. Thus, ROS inhibition decreased autophagy and apoptosis in NSCLC cell lines (173).

Autophagy, as a response to treatment, is diverse. Cytotoxic autophagy is characterized by promotion of cell death associated with apoptosis and reduced sensitivity to treatment when it is inhibited (159). Rituximab-monomethyl auristatin E (MMAE) treatment, induced cell death by autophagy in B cell lymphoma by inactivating the AKT/mTOR pathway. Cell death was stimulated with exposure to rapamycin and was inhibited with chloroquine (174). Oridonin is an active diterpenoid compound isolated from *Rabdosia rubescens*. Colorectal carcinoma lines HT-29, HCT116, SW480, and S620 exposed to oridonin showed autophagic cell death due to metabolic imbalance characterized by a dramatic inhibition of glucose uptake without reduction of ATP levels. In this setting, tumor cells become autophagy-dependent to meet energetic and nutritional demands to sustain viability, causing autophagic cell death (175). Brefeldin A is a lactone that inhibits protein transport from the endoplasmic reticulum to the Golgi apparatus. In colorectal carcinoma cell lines and xenograft tumor models, brefeldin A produced stress at the endoplasmic reticulum level by increasing regulation and interaction of binding immunoglobulin protein (Bip) with AKT, which activated autophagic cell death (176). In other study, when folate receptor was blocked using a monoclonal antibody MORAB-003 (farletuzumab) in ovarian cancer cells and in an orthotopic mouse models tumor growth was inhibited due to autophagic cell death. When MORAB-003 was combined with hydroxychloroquine, the inhibition of tumor growth was reversed (177).

Chloroquine and hydroxychloroquine are the only autophagy inhibitors approved by the FDA for clinical use (178), comprehensive reviews are examining the role of various compounds and biological molecules in the regulation of autophagy and various ATG genes (160, 176, 179–181). Clinical trials are underway in which inhibitors of autophagy are administered in combination with chemotherapy or targeted therapy (182, 183). However, because of dissimilar participation of autophagy as a cytoprotective or cytotoxic mechanism, biomarkers related to these scenarios must be identified to predict treatment response.

## Concluding Remarks

The role of autophagy in several stages of tumor development is reviewed. Metabolic status through distinct stages of tumor impacts in tumor suppressor or tumor-promoting roles of autophagy is discussed. In incipient tumors, nutrient, and oxygen supply is sufficient and do not represent environmental stress; therefore, autophagy acts as an intrinsic cytotoxic response suppressing tumor development. However, as tumor grows metabolic requirements are increased to sustain high proliferation rates. Autophagy provides reduced carbon to maintain the energy demand and support survival of tumor cells in hostile microenvironments. In advanced stages of tumor development, the hypoxic, and starvation conditions generate signals promoting tumor invasion and metastasis. Autophagy helps cells evade anoikis and promote focal adhesion turnover favoring cell motility and metastasis. Additionally, autophagy serves as an immune evasion strategy in cancer advanced stages. In these settings, autophagy might promote resistance to chemotherapy or targeted therapy in most scenarios.

We consider autophagy and cancer metabolism parts of an overall process. For this reason, it is necessary to consider the metabolic status of tumor for use of autophagy inhibitors as a therapeutic strategy for impacting clinical outcomes.

## Author Contributions

DA-C, RC-D, and MP-M organized the entire manuscript, wrote the draft, and revised the last version of the manuscript. RC-D and JL-G wrote the autophagy, apoptosis crosstalk, and involvement of autophagy in tumor immune evasion. MP-M and DA-C wrote the autophagy and cancer metabolic reprograming section. DA-C and MG-V wrote the interplay of autophagy in metastasis. RC-D and JL-G wrote involvement of autophagy in tumor immune evasion. DA-C and JL-G wrote autophagy in chemotherapy and target therapy resistance. Figures 1, 2, 5 were designed by RC-D. Figure 3 was designed by MP-M and Figure 4 by DA-C. All authors contributed to the article and approved the submitted version.

## Conflict of Interest

The authors declare that the research was conducted in the absence of any commercial or financial relationships that could be construed as a potential conflict of interest.
